# Levels of 8-OxodG Predict Hepatobiliary Pathology in *Opisthorchis viverrini* Endemic Settings in Thailand

**DOI:** 10.1371/journal.pntd.0003949

**Published:** 2015-07-31

**Authors:** Prasert Saichua, Anna Yakovleva, Christine Kamamia, Amar R. Jariwala, Jiraporn Sithithaworn, Banchob Sripa, Paul J. Brindley, Thewarach Laha, Eimorn Mairiang, Chawalit Pairojkul, Narong Khuntikeo, Jason Mulvenna, Paiboon Sithithaworn, Jeffrey M. Bethony

**Affiliations:** 1 Biomedical Science Program, Faculty of Graduate School, Khon Kaen University, Khon Kaen, Thailand; 2 Department of Preclinical Science, Faculty of Medicine, Thammasat University, Pathumthani, Thailand; 3 Department of Microbiology, Immunology and Tropical Medicine, and Research Center for Neglected Diseases of Poverty, School of Medicine & Health Sciences, George Washington University, Washington, D.C., United States of America; 4 Department of Clinical Microscopy, Faculty of Associated Medical Sciences, Khon Kaen University, Khon Kaen, Thailand; 5 Department of Pathology, Faculty of Medicine, Khon Kaen University, Khon Kaen, Thailand; 6 Liver Fluke and Cholangiocarcinoma Research Center, Faculty of Medicine, Khon Kaen University, Khon Kaen, Thailand; 7 Department of Parasitology, Faculty of Medicine, Khon Kaen University, Khon Kaen, Thailand; 8 Department of Radiology, Faculty of Medicine, Khon Kaen University, Khon Kaen, Thailand; 9 Department of Surgery, Faculty of Medicine, Khon Kaen University, Khon Kaen, Thailand; 10 Infections Disease Program, QIMR Berghofer Medical Research Institute, Brisbane, Queensland, Australia; McGill University, CANADA

## Abstract

*Opisthorchis viverrini* is distinct among helminth infections as it drives a chronic inflammatory response in the intrahepatic bile duct that progresses from advanced periductal fibrosis (APF) to cholangiocarcinoma (CCA). Extensive research shows that oxidative stress (OS) plays a critical role in the transition from chronic *O*. *viverrini* infection to CCA. OS also results in the excision of a modified DNA lesion (8-oxodG) into urine, the levels of which can be detected by immunoassay. Herein, we measured concentrations of urine 8-oxodG by immunoassay from the following four groups in the Khon Kaen Cancer Cohort study: (1) *O*. *viverrini* negative individuals, (2) *O*. *viverrini* positive individuals with no APF as determined by abdominal ultrasound, (3) *O*. *viverrini* positive individuals with APF as determined by abdominal ultrasound, and (4) *O*. *viverrini* induced cases of CCA. A logistic regression model was used to evaluate the utility of creatinine-adjusted urinary 8-oxodG among these groups, along with demographic, behavioral, and immunological risk factors. Receiver operating characteristic (ROC) curve analysis was used to evaluate the predictive accuracy of urinary 8-oxodG for APF and CCA. Elevated concentrations of 8-oxodG in urine positively associated with APF and CCA in a strongly dose-dependent manner. Urinary 8-oxodG concentrations also accurately predicted whether an individual presented with APF or CCA compared to *O*. *viverrini* infected individuals without these pathologies. In conclusion, urinary 8-oxodG is a robust ‘candidate’ biomarker of the progression of APF and CCA from chronic opisthorchiasis, which is indicative of the critical role that OS plays in both of these advanced hepatobiliary pathologies. The findings also confirm our previous observations that severe liver pathology occurs early and asymptomatically in residents of *O*. *viverrini* endemic regions, where individuals are infected for years (often decades) with this food-borne pathogen. These findings also contribute to an expanding literature on 8-oxodG in an easily accessible bodily fluid (e.g., urine) as a biomarker in the multistage process of inflammation, fibrogenesis, and infection-induced cancer.

## Author Summary

*Opisthorchis viverrini* is a food-borne helminth infection that drives a strong inflammatory response in the bile duct that can result in bile duct fibrosis and bile duct cancer (intrahepatic cholangiocarcinoma). Extensive research shows that oxidative stress (OS) plays a critical role in chronic *O*. *viverrini* infection transitioning to cancer in the bile duct. OS also results in a modified DNA lesion, referred to as 8-oxodG, excreted in the urine, where it can be detected by an antibody-based test. We measured the concentrations of 8-oxodG in the urine of *O*. *viverrini*-infected individuals who had developed bile duct fibrosis or bile duct cancer and compared levels of this metabolite in urine to *O*. *viverrini* infected individuals who did not have bile duct fibrosis or cancer in Northeastern Thailand. We determined bile duct fibrosis by ultrasonography and bile duct cancer by immunohistochemistry on resected liver tissue. We then built a statistical model to quantify how well urinary 8-oxodG predicted bile duct fibrosis and bile duct cancer in *O*. *viverrini*-infected individuals. We found that individuals with elevated levels of 8-oxodG in urine had a greater probability of developing bile duct fibrosis or bile duct cancer from *O*. *viverrini* infection. This association occurred in a strongly dose-dependent manner: in other words, the *O*. *viverrini*-infected individuals who had the highest concentration of urinary 8-oxodG also had the highest risk of presenting with bile duct fibrosis or bile duct cancer. In summary, measuring levels of 8-oxodG in the urine offers a unique opportunity to develop a candidate biomarker for advanced *O*. *viverrini* induced hepatobiliary pathologies such as fibrosis and cancer. The findings also confirm our previous observations that severe liver pathology occurs early and asymptomatically in residents of *O*. *viverrini* endemic regions, where individuals are infected for years (often decades) with this food-borne neglected tropical diseases (NTD) pathogen.

## Introduction

Over 750 million people (10% of the human population) are at risk of infection with food-borne trematodes, with more than 40 million people currently infected with one of three of these parasites: *Clonorchis sinensis*, *Opisthorchis felineus*, *and Opisthorchis viverrini* [1, 2]. *O*. *viverrini* is considered the most important of these food-borne trematodes due to its well-documented association with hepatobiliary pathologies that include advanced periductal fibrosis (APF) [3, 4] and intrahepatic cholangiocarcinoma (CCA) [5–10]. In Northeastern Thailand (Isaan), uncooked cyprinoid fish, which is the intermediate host for the parasite, are a staple of the diet, with *O*. *viverrini* infecting an estimated 10 million people in Isaan alone [8]. While infection with *O*. *viverrini* can be eliminated by chemotherapy (praziquantel), regional culinary practices result in rapid re-infection after treatment, often leading to life-long infection with the parasite [5, 7, 11] and the highest incidence of CCA in the world (85 per 100,000) [7].

In our community-based ultrasound studies in Northeastern Thailand [8, 9, 12, 13], we have identified a series of pathologic changes that occur early and asymptomatically in the bile duct in individuals resident in *O*. *viverrini* endemic areas. As individuals can be infected with *O*. *viverrini* for years (even decades), we hypothesize that a chronic cycle of tissue damage and repair ensues in the intrahepatic biliary ducts as a result of the constant immunological, mechanical and oxidative damage from the parasite, resulting in a persistent “smoldering and chronic inflammatory milieu” [14]. These processes stimulate the production of desmoplastic stroma (i.e., bile duct fibrosis), which has recently been shown to play a crucial role in promoting malignant transformation to CCA (see [15]). In both the hamster and human models of *O*. *viverrini* infection, fibrosis in the biliary epithelia routinely precedes CCA [8, 10, 16, 17].

The exact mechanism by which stromal desmoplasia (bile duct fibrosis) transforms to CCA is a topic of intense research [15, 17–19]. There is some consensus that an important component in this transformation is the genomic instability that accompanies both fibrogenesis and carcinogenesis[20, 21]. During these processes, cells are recruited to the site of damage and induce a “respiratory burst” from an increased uptake of oxygen, with the accumulation of reactive oxygen species (ROS) referred to as oxidative stress (OS) (see [22]). Both fibrotic [16] and neoplastic transformation [20, 23] have been linked to increased levels of OS by several mechanisms, including DNA damage, genomic instability, and cellular proliferation, The DNA base modifications caused by OS also result in oxidation of guanine residues to 8-oxo-7,8-dihydro-2′-deoxyguanosine (8-oxodG), which are excised into bodily fluids, such as urine, blood and saliva. Augmented 8-oxodG levels in urine have been used as a biomarker for oxidative DNA damage [20, 21, 24–28] in acute lymphoid leukemia, colorectal cancer, high grade cervical dysplasia, scleroderma fibrosis, liver fibrosis, renal cell carcinoma, lung cancers, and prostate cancer [15, 22, 29, 30].

Elevated levels of 8-oxodG have been reported in the urine of individuals chronically infected with *O*. *viverrini* and in the urine of individuals with *O*. *viverrini*-induced CCA [31–33]. However, to our knowledge, there have been no studies that measure levels of urine 8-oxodG during the interval from initial infection with *O*. *viverrini* to neoplastic transformation to CCA, a progression which occurs over years and proceeds through several well-defined hepatobiliary pathologies [12]. The most precisely detected and well documented of these hepatobiliary pathologies is APF [9]. Our community-based ultrasound studies (i.e., Khon Kaen Cancer Cohort or the KKCC) along the Chi River, Khon Kaen Province, Thailand have shown that APF is prevalent among otherwise apparently healthy residents in *O*. *viverrini* endemic areas [5, 8–10, 13]. The objective of the current manuscript was to determine if levels of 8-oxodG in urine increased when *O*. *viverrini* infection transitioned to APF [34] and if elevated levels of urine 8-oxodG associated with APF were comparable to the markedly elevated levels of urine 8-oxodG observed in individuals with *O*. *viverrini*-induced CCA [17, 28, 35]. As APF is a precursor stage to CCA, a threshold concentration of urinary 8-oxodG could then be used to risk stratify APF individuals into those more likely to develop *O*. *viverrini*-induced CCA and, as such, take advantage of recent therapeutic advances used to target bile duct fibrosis and the rich milieu it provides for neoplastic transformation to CCA [36]. More specifically, our objectives were as follows: (a) quantify the presence of urinary 8-oxodG in *O*. *viverrini* infected individuals with APF compared to *O*. *viverrini*-infected individuals without APF and *O*. *viverrini*-individuals with CCA and (b) determine the performance of urine 8-oxodG in predicting APF, including its sensitivity, specificity, and ability to predict diagnostic risk (e.g. odds ratios). These studies form the “discovery phase” for a potentially critical and easily accessible candidate biomarker that could be advanced to biomarker validation in large-scale studies in Thailand and in the neighboring countries of the Mekong River Basin, where *O*. *viverrini*-induced CCA has the highest incidence in the world [7, 12].

## Materials and Methods

### Ethics statement

The participants provided written informed consent using forms approved by the Ethics Committee of Khon Kaen University School of Medicine, Khon Kaen, Thailand (reference number HE480528) and the Institutional Review Board (IRB) of the George Washington University School of Medicine, Washington, D.C (GWUMC IRB# 020864). The urine donated by participants from Group 4 was obtained from the biological specimen repository of the Liver Fluke and Cholangiocarcinoma Research Center, Khon Kaen University, Thailand using a protocol approved by the Ethical Committee on Human Research, Faculty of Medicine, Khon Kaen University, Khon Kaen, Thailand (reference Nos. HE450525 and HE531061).

### Study population and description of inclusion and exclusion criteria

**Fig 1:**
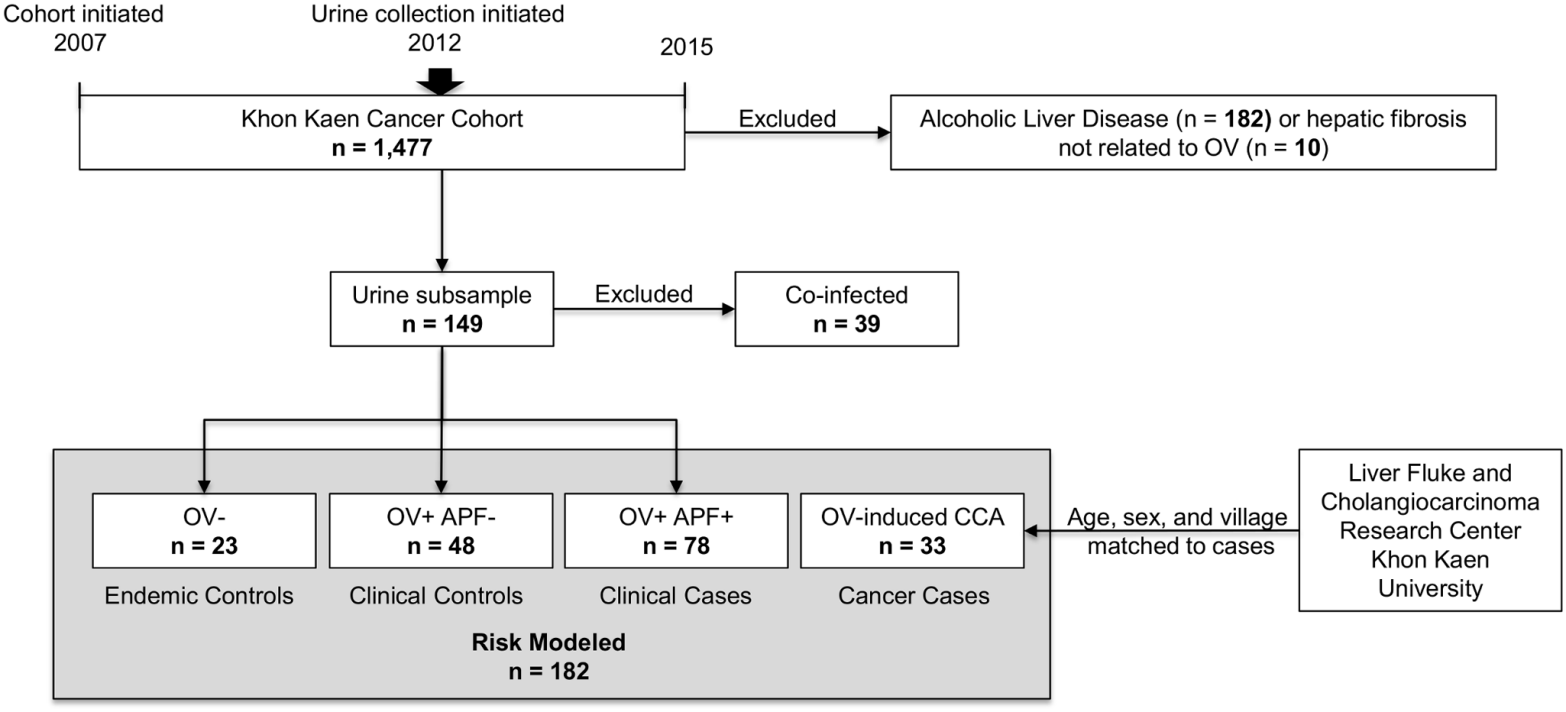
A schematic representation of study participants from Khon Kaen Cancer Cohort (KKCC) and the Liver Fluke and Cholangiocarcinoma Research Center. The KKCC is identified along with approved modifications to the protocol. The exclusion criteria used at various stages are also presented. The stratification of participants into the groups used in the analyses of levels of urine 8-oxodG are depicted.

This study uses baseline data from a recently enrolled village in the Khon Kaen Cancer Cohort (KKCC), which samples villages along the Chi River Basin, in Khon Kaen province, Thailand. A detailed description of the KKCC and the methods used to assemble this cohort have been extensively reported [8–10, 13]. A modification to the original KKCC protocol was approved by the Division of Microbiology and Infectious Diseases (DMID) of the US National Institutes of Health, the Ethics Committee of Khon Kaen University School of Medicine, and the GWU IRB, allowing urine collection, which initiated in 2012 (Fig 1). The current sample of 221 individuals represents the most recent enrollment into the KKCC following this modified protocol (Fig 1). Participant inclusion criteria consisted of enrolling all males and females between 20 and 60 years of age (inclusive) registered with the village health outpost who were willing to participate in the study as evidenced by signing the informed consent form. Participants were excluded from the study if they attended school or worked full-time outside of the village (n = 0) or had a positive urine β-hCG pregnancy test in the case of females (n = 0). Individuals infected with *O*. *viverrini* were referred to the local public health clinic for treatment with praziquantel (PZQ) regardless of their participation in the study.

The data for the 181 KKCC participants were stratified during analyses to remove thirty two (n = 32) individuals whose feces contained eggs or larvae from other helminths endemic to the region, including hookworm (*Necator americanus*), *Ascaris lumbricoides*, or *Strongyloides stercoralis* (Fig 1). This reduced the study sample to 149 individuals assigned to three groups as follows: Group 1 (n = 23) individuals negative (uninfected) upon fecal examination for *O*. *viverrini* (also referred to as Endemic Controls or EN); Group 2 (n = 48) *O*. *viverrini* positive (OV+) individuals who were negative for APF as determined by US (also refereed to as Clinical Controls); and Group 3 (n = 78) *O*. *viverrini* positive individuals who were also positive for APF as determined by US (also referred to as Clinical Cases) (Fig 1). Group 4 consists of samples from 33 individuals who were not part of the KKCC, but had histologically proven *O*. *viverrini*-associated CCA, whose urine samples were stored at the biological specimen repository of the Liver Fluke and Cholangiocarcinoma Research Center, Khon Kaen University, Thailand. The samples from Group 4 were chosen from the biological specimen repository by matching them on age, sex, and nearest neighbor (i.e., residence at time of death in a village within 10 kilometers of the current study sample) to the Clinical Cases in the KKCC.

### Abdominal ultrasound for the detection of advanced periductal fibrosis (APF)

A detailed description of the ultrasonography methods used in this study can be found in the following references [8–10]. Briefly, a mobile, high-resolution ultrasound (US) machine (GE model LOGIQ Book XP) was used. Hepatobiliary abnormalities including portal vein radical echoes, echoes in liver parenchyma, indistinct gallbladder wall, gallbladder size, sludge and suspected CCA were graded and recorded. Individuals were classified as “Non-Advanced Periductal Fibrosis” (APF-) or “controls” if the US grade was 0 or 1, and “Advanced Periductal Fibrosis” (APF+) or “case” if the US grade was 2 or 3. Individuals with alcoholic liver disease, which is seen as fatty liver by US exam, were excluded from this study. Also, individuals with marked hepatic fibrosis not related to OV infection (e.g., cirrhosis from HBV or HCV) were also excluded from this study (see Fig 1).

### Collection of samples and their clinical assessment

#### Collection of feces and urine from participants

Samples of feces and of urine were provided on the same day by each participant in Groups 1–3. Fecal samples were not available for participants in Group 4 (CCA cases) but *O*. *viverrini* status was noted in their clinical records at the time of death. Diagnosis of infection with *O*. *viverrini* was established and quantified as eggs per gram of feces (epg) by microscopic examination of feces using the formalin-ethyl acetate concentration technique (FECT), as described in [37] with duplicate examinations of fecal samples.

First morning, mid-stream, urine samples were collected into sterile containers. Unprocessed urine was screened for urinary tract infection by urine strip (ARKRAY’s AUTION Sticks, Japan) and analyzed by an Automated Urine Chemistry Analyzer (AUTION MAX AX-4280, Arkray, USA). Specimens which showed abnormal values were excluded from the study i.e. leukocyte or nitrate positive. Urine samples were centrifuged and the supernatant aliquoted and stored at −20°C. Urinary creatinine concentrations were determined by the Jaffe method [38].

For Group 4 (cases of CCA), urine was age, sex, and nearest-neighbor-matched (by village) using specimens in the repository of the Liver fluke and Cholangiocarcinoma Research Center, Khon Kaen University, Thailand. Participants had been asked to collect first morning urine into clean polypropylene containers, which was held on ice until processed by centrifugation in the laboratory, aliquoted and stored at −20°C.

#### Collection of serum from study participants

Thirty milliliters of venous blood were collected from individuals in Groups 1, 2 and 3, into siliconized tubes after overnight fasting. Venous blood samples were allowed to clot at room temperature for 30 minutes after collection, centrifuged, and the serum recovered, aliquoted for storage at −20°C in a temperature-monitored freezer. Serum was not available for participants in Group 4 (CCA cases).

#### Measurement of 8-oxodG in urine

Concentration of 8-oxodG in urine was measured using an HT Trevigen (Gaithersburg, MD) validated competitive ELISA kit. This assay quantifies 8-oxodG in urine using the anti-8-oxodG monoclonal antibody clone 2E2. Briefly 150 μl of 8-OHgG (8-hydroxy-2’-deoxyguanosine) standard were added in triplicate starting at a concentration of 200 nM (56.7 ng/ml) and serially diluted using 1X in phosphate buffered saline (pH 7.2) with 0.1% Tween 20 (PST20) to 3.3 nM (0.89 ng/mL) onto a 96 well microtiter plates pre-coated with 8-oxodG, followed by duplicates of experimental urine samples diluted at 1:20 in PST20. Blank wells with 150 μl of PBST20 and 8-OHgG alone were added in triplicate to each plate as assay controls. Subsequently, 25 μl of monoclonal mouse IgG2b anti-8-oxodG (clone 2E2) conjugated to Keyhole Limpet Hemacyanin (KLH) and horseradish peroxidase (HRP) were diluted in PBST20 and added to each well. The monoclonal mouse IgG2a anti-8-oxodG (clone 2E2), conjugated to KLH specifically binds to 8-hydroxy-2'- deoxyguanosine within DNA as described [39]. The plate was covered and incubated for 1 hour (HR) at room temperature (RT) in the dark. The plates were then washed 6 times using 300 μl/well of 1X PBST20 (7.2 pH) and 100 μl of 3,3’,5,5’-Tetramethylbenzidine (TMB) substrate was added to each well and incubated at RT for 15 min in the dark. The reaction was stopped by adding 50 μl 0.2M HCL to the well. Optimal density at 450 nanometers (nm) was determined on a SpectraMax 340PC384 system (Molecular Devices, Sunnyvale CA) using SoftMax Pro Software (Molecular Devices, Sunnyvale CA) for data management. The standard curve was estimated using a polynomial function and level of 8-oxodG in urine were interpolated onto this standard curve and expressed in ng/ml.

#### Standardization of 8-oxodG by creatinine concentration in urine as determined by the Jaffe method

Measures of creatinine in the urine samples of study participants were determined in ng/mL by the Jaffe method as described above [38]. To standardize the levels of 8-oxodG across participants, level of 8-oxodG was divided by urine creatinine to obtain an adjusted measure of 8-oxodG (in ng/mg of creatinine) [40]. Levels of creatinine were unavailable for 37 individuals in the study. When creatinine levels were unavailable (e.g. for urine from CAA patients), the levels of 8-oxodG were estimated as follows: individuals were separated by sex and infectivity status and their average creatinine concentrations estimated. The creatinine averages were used to adjust the 8-oxodG levels as described above.

#### Description of the enzyme-linked immunosorbent assay (ELISA) protocol used to quantify levels of the antibody against *O*. *viverrini* antigen in serum

The ELISA protocol for serum IgG antibody measurements has been previously described in detail [13]. Briefly, flat-bottom 96 wells microtiter plate (NUNC, DN) were coated overnight at 4°C with 1 μg/ml of crude somatic *O*. *viverrini* antigen in a PBS buffer. After washing with a buffer that contained 0.05% Tween 20 in PBS (PBST20), a blocking buffer containing 3 w/v BSA (Fitzgerald, MA) in PBS and 0.5% Tween 20 (Fisher, NJ) was added and incubated at RT for 1 HR. The diluted serum samples were added in duplicate at 100 μL/well, covered, and incubated overnight at 4°C. After washing with PBST20, 100 μL of a horseradish peroxidase conjugated goat anti-human IgG (Zymed, CA) diluted in PBST20 was added at 1:1000 and incubated for 2 HR at RT. After incubation and washing with PBST20, the substrate solution which consisted of Ortho-phenylenediamine (Sigma, MO), 53 mM citric acid anhydrous (Fisher, NJ), 102 mM diasic sodium phosphate dodecahydrate (Acros Organics, NJ) and 30% w/w of hydrogen peroxide (Fisher, NJ) in DI water was added to the plates and incubated at RT in the dark for 30 min. The reaction was stopped by 2N sulfuric acid (BDH, PA) and plates were read using a SpectraMax 340PC^384^ microplate reader (Molecular Devices Sunnydale, CA) at 492_nm_. A standard calibration curve (SCC) made from a pool of high titer human sera against the same crude somatic *O*. *viverrini* adult antigen extract was run on each plate in doubling dilutions starting at 1:200 to form a sigmoidal curve when modeled by a 4-parameter logistic (4-PL) using Softmax Pro software (Molecular Devices, Sunnydale, CA). Homologous interpolation between the experimental serum samples and SCC were then used to determine the levels of IgG against the OV crude antigen extract expressed in Arbitrary Units as discussed in detail in Sachiua et al. [13].

### Statistical analysis

Data analysis was performed with SAS 9.2 (SAS Institute Inc., Cary, NC, USA). The raw data used to generate this manuscript are publicly available from the Dryad Digital Repository with the accession number doi:10.5061/dryad.pd6mn.

#### Characteristics of study participants

Descriptive statistics, including number of observations (n), mean, median, standard deviation (SD), and 95% confidence intervals (95% CI) were calculated for demographic variables and baseline characteristics of study participants (Table 1). In instance were a group had less than 5 observations only the median was obtain and reported. The Kruskal-Wallis one-way analysis of variance was used to test for differences in levels of urinary 8-oxodG in study participants stratified by the groups described above (EN, OV+APF-, OV+APF+, or CCA) [41] and to obtain p values. In instances where the test produced a significant result, Dunn's multiple comparisons procedure was used to perform pair-wise comparisons of distributions of urinary 8-oxodG levels between the study groups (family-wise error rate was controlled at 5%).

**Table 1:** Creatinine adjusted levels of urine 8-oxodG in the study sample.

	Median	Mean	SD	95%CI	N	P value^Ɨ^
**Sex**						
**Males**	160.17	247.73	235.37	197.86–297.60	88	0.86
**Females**	148.33	216.16	193.95	176.44–255.89	94	
**Age strata**						
**20–29** ^a^	171.40				3	0.43
**30–39**	201.66	248.87	240.38	144.92–352.82	23	
**40–49**	132.46	218.71	221.70	162.41–275.01	62	
**50+**	149.97	235.88	208.80	193.12–278.65	94	
**Smoking Status** ^b^						
**No**	147.55	201.59	184.30	164.04–239.13	95	0.21
**Yes**	185.93	270.05	245.28	213.62–326.48	75	
**Alcohol Consumption** ^c^						
**No**	133.43	176.18	147.62	126.69–225.40	37	0.23
**Yes**	154.83	237.46	224.40	195.25–279.67	111	
**Diagnostic Criteria**						
**EN (OV-)**	103.72	116.18	54.62	92.56–139.80	23	**<0.0001**
**OV+/APF-**	126.10	143.77	120.61	108.75–178.80	48	
**OV+/APF+**	236.08	300.41	245.99	244.94–355.87	78	
**CCA**	179.77	276.19	239.98	191.10–361.28	33	

OV refers to *O*. *viverrini*. APF refers to advanced periductal fibrosis. CCA refers to cholangiocarcinoma.

^a^ Mean, standard deviation (SD), and 95% confidence interval (CI) not reported when less than 5 participants.

^b^ Smoking status not available for 12 participants.

^c^ Alcohol consumption not available for 34 participants.

^Ɨ^ Kruskal-Wallis ANOVA used to determine significant differences in levels of urinary 8-oxodG among groups and compute the p values. Significant p-values in bold font.

#### Description of the strategy used to assemble the urine 8-oxodG model for corroborating diagnosis with *O*. *viverrini* associated pathology

A risk model [42] initially containing potentially relevant demographic [11, 37], behavioral [37, 43], and immunological [6, 44–48] markers was constructed to evaluate the utility of the level of urinary 8-oxodG in predicting APF and CCA status. Our previous reports of elevated levels of serum IgG and IgG1 in individuals with medium to heavy *O*. *viverrini* infections prompted us to include serum IgG and IgG1 to *O*. *viverrini* crude adult antigen extract as covariates in the biomarker model along with levels of 8-oxodG in the initial stages of model assembly [48]. Age, alcohol consumption (reported and analyzed as a dichotomous variable), and smoking status (reported and analyzed as a dichotomous variable) were also considered as possible covariates [11, 43, 45]. The logistic regression procedure was applied in two parts: in the first phase the model was fit to the data to compute the probability (odds) of receiving a diagnosis of either APF or CCA (categorical) [42], followed by models specifically considering the relationships between EN/APF- participants and either APF+ or CCA participants. The initial model included levels of urinary 8-oxodG, serum levels of IgG and IgG1, and the participants' age, and alcohol consumption habits (equation 1 provided in S1 Text—Supplementary Equations and Definitions). Smoking status was not initially modeled, because the information was not available for CCA participants.

We applied stepwise selection, with backward elimination of predictors, to the full five-predictor model. An initial standard selection criterion of p ≤ 0.05 was used for inclusion of a variable in the model [42, 49]. The backward elimination procedure was initially used to eliminate non-significant predictor variables (p > 0.05) from the model that included all three diagnostically distinct disease levels. Subsequent to this initial modeling step, separate logistic regression models were considered to compare the control individuals with either the APF+ individuals or the CCA individuals. These models included predictors that were previously identified as significant and were re-fitted with the step-wise selection procedure and backward elimination to identify the predictors that remained significant for these specific diagnostic models. During this second round of model fitting, predictors with p-values below 0.05 were considered significant and were retained in the model. The final parsimonious models included urinary 8-oxodG as the only significant predictive biomarker. The models are described fully in the results section as well as in equation 2 provided in S1 Text—Supplementary Equations and Definitions.

Odds Ratios (OR) were obtained from these risk models; the OR from the risk model for CCA were adjusted for age.

#### Elucidating diagnostically informative biomarker levels from the urinary 8-oxodG risk models

Receiver operating characteristic (ROC) curves [49] were constructed by plotting the sensitivity against 1-specificity for the entire range of the possible creatinine adjusted urinary 8-oxodG values of the study participants. Since the ROC curves capture the entire range of the possible levels of urinary 8-oxodG, it was possible to examine a variety of potential cutpoints, or threshold levels, of the biomarker to identify diagnostically meaningful levels, which may prove relevant in a clinical setting. Sensitivity and specificity values were estimated by the logistic regression procedure for all of the possible cutpoints identified from the ROC curve. Cutpoints that produced simultaneously maximized values of sensitivity and specificity were identified as 'optimal' by the model and were subsequently considered informative and clinically relevant. These threshold values were used to cross-classify the observed and predicted responses of study participants to generate probabilities associated with receiving a positive diagnosis of APF or CCA. Sensitivity and specificity values were estimated from the probabilities by the logistic regression procedure and were subsequently used to evaluate the validity of the diagnostic assay.

#### Assessing the accuracy of the diagnostic assay, which utilizes informative threshold levels of urinary 8-oxodG, for potential use in the clinical setting

The performance of the diagnostic assay was evaluated by computing the positive predictive value (PPV), negative predictive value (NPV), the likelihood ratio of obtaining a positive test result (LR+), and the likelihood ratio of obtaining a negative test result (LR-). These values were calculated for each relevant diagnostic positivity threshold, using a 50% prevalence rate of *O*. *viverrini* infection, as determined by fecal exam reported in our previous studies [8–10, 13], in adults between 20 and 60 years of age (inclusive).

The formulae used to calculate measures of assay performance (PPV and NPV) when used in an *O*. *viverrini*-endemic area [50], as well as the definitions of the LR+ and LR- are included in the supplementary text (Equations 3 and 4 in S1 Text—Supplementary Equations and Definitions).

## Results

### Descriptive statistics for urinary 8-oxodG in the study sample

Table 1 shows the descriptive statistics for urinary 8-oxodG levels by sex, age strata, smoking status, alcohol consumption, and the criteria used to diagnose various stages of *O*. *viverrini*-induced infection. The levels were highest among persons in the 30–39 years of age group followed by individuals in the 20–29 years of age group, which consisted of only three individuals. Smokers had higher median levels of 8-oxodG at 185.93ng/mg creatinine compared to 147.55ng/mg creatinine in non-smokers. Similarly, individuals who indicated that they consumed alcohol had higher median levels of creatinine adjusted 8-oxodG compared to those who did not, with 154.83ng/mg creatinine and 133.43ng/mg creatinine, respectively. The 8-oxodG levels increased with advancing disease status.

### Creatinine adjusted 8-oxodG levels were significantly elevated in individuals with more advanced hepatobiliary disease

The Kruskal-Wallis one-way analysis of variance demonstrated that there were significant differences in the distributions of urinary 8-oxodG levels between participants separated into groups by disease progression (p < .0001). Dunn's multiple comparisons procedure, for which the family-wise error rate was set at 0.05, identified significant (p < 0.05) differences in the distributions of urinary 8-oxodG between control individuals (both EN and APF-) and *O*. *viverrini*-infected individuals (APF or CCA positive). No significant differences were observed between participants in EN and APF- groups, which, along with co-occurrence of these individuals in the same population, justified combining these participants into one group in subsequent analyses. No significant difference was observed between participants in the APF+ and CCA groups. Lack of statistically significant findings here may be explained by the presence of three individuals in the APF+ group who had extremely high levels of urinary 8-oxodG (4.66, 4.33, and 4.04 times greater than the group's median urinary 8-oxodG levels). However, the groups remained distinct in subsequent analyses due to the presence of the outlier values and also due to the specific pathological differences that exist between APF+ and CCA individuals.

**Fig 2:**
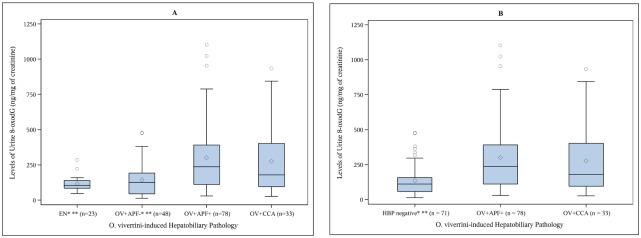
Levels of creatinine corrected urinary 8-oxodG are significantly elevated in *O*. *viverrini* infected individuals with advanced periductal fibrosis (APF) and *O*. *viverrini* induced intrahepatic cholangiocarcinoma (CCA). The distributions of 8-oxodG levels in urine are shown in Panel A for all four groups and in Panel B for three groups, with the first group in Panel B being a combination of Endemic Normal (*O*. *viverrini* negative) and the APF- participants into a single hepatobiliary negative control group. The length of the boxes represents the interquartile range (IQR) or the distance between the 25th and 75th percentiles of each urinary 8-oxodG study group. The median value of each urinary 8-oxodG group are represented by the solid horizontal line in each box; the mean value of the group is depicted by the diamond character in the boxes. The bottom whisker represents the value of the lowest observation in the group. The highest observations in the group are represented by the uppermost circles above the group box; the upper whiskers represents the highest observations within 1.5x IQR. ^a^Significant difference exists between this group and the APF+ individuals; this result was obtained using Dunn's procedure for multiple comparisons following the Kruskal-Wallis one way analysis of variance. ^b^Significant difference exists between this group and the CCA individuals; this result was obtained using Dunn's procedure for multiple comparisons following the Kruskal-Wallis one way analysis of variance.

Fig 2 illustrates the distribution of urinary 8-oxodG in individuals participating in this study. Panel A of Fig 2 depicts the distributions of the levels of urinary 8-oxo-dG in each of the four groups of the study participants and Panel B illustrates the distributions following the amalgamation on participants from groups 1 and 2. Superscripts above the group labels on the x-axis indicate significant findings. Following the combination of control participants into one group (HBP negative), the following results were obtained: the concentrations of 8-oxodG in both cases and controls ranged between 14.25ng/mg creatinine and 1100.97ng/mg creatinine. The range was narrower in the control group (14.25 to 476.58 ng/mg creatinine, n = 71) than among cases, especially OV+ and APF+ (28.89 to 1100.96 ng/mg creatinine, n = 78) and OV+/CCA individuals (26.74 to 933.77 ng/mg creatinine, n = 33). The median 8-oxodG levels of persons with APF or CCA were significantly higher (2.12x and 1.62x, respectively) than the median level of the control group as determined by Dunn's multiple comparisons procedure applied to the Kruskal-Wallis one-way analysis of variance, with the family-wise error rate set at 0.05. These data indicated progressively higher levels of 8-oxodG in the urine during advanced hepatobiliary disease during opisthorchiasis.

### Elevated levels of creatinine-adjusted urinary 8-oxodG indicate a significantly increased risk of diagnosis with advanced periductal fibrosis and cholangiocarcinoma in a dose-dependent manner

Various candidate biomarkers, including urinary 8-oxodG, serum level of IgG and IgG1 against a crude adult *O*. *viverrini* antigen extract were modeled as potential relevant predictors of progression to advanced hepatobiliary pathology during chronic *O*. *viverrini* infection. In the initial stages of model development, control individuals, APF+ individuals, and CCA individuals were included in the model to identify the potential relevant predictors of disease progression. This model identified age (p = 0.0065), serum IgG (p = 0.0002), and urinary 8-oxodG (p < 0.0001) as significant and these predictors were subsequently considered in the two models comparing control individuals to either APF+ participants or CCA participants. In this second stage of model development, urinary 8-oxodG was retained as the single significant biological predictor of an individual having ultrasound confirmed APF (*p* ≤ 0.0001) or histologically confirmed CCA (*p* ≤ 0.0014)). Age and serum IgG were no longer significant in the model of APF+ participants (p = 0.4802 and p = 0.2890, respectively). In the model of CCA individuals age remained a significant predictor (p < 0.0001), while serum IgG was eliminated as non-significant (p = 0.0587). The final parsimonious models retain the concentration of creatinine-adjusted urinary 8-oxodG as the only predictive biomarker (for diagnosis of both APF and CCA) along with age as a significant covariate in individuals diagnosed with CCA (equation 2 in S1 Text—Supplementary Equations and Definitions).

The results of a logistic regression model that included urinary 8-oxodG as a predictor were considered alongside the actual ability of the clinical (gold standard) methods to identify specific increases in the level of 8-oxodG in urine of participants in the study. Increasing odds ratios (ORs) were associated with progressively higher concentrations of 8-oxodG, indicting the increased likelihood of progressing to APF and CCA for individuals with higher measurable concentrations of creatinine-adjusted 8-oxodG in the urine. The ORs and their 95% confidence intervals (95% CIs) are summarized in Table 2.

**Table 2:** Progressively higher urinary 8-oxodG levels show an increasing risk of having received a clinical diagnosis of APF+ or CCA status in *Opisthorchis viverrini* infected individuals.

	APF Status		CCA Status	
Urine 8-oxodG (ng/mg creatinine)	OR	95% CI	aOR*	95% CI*
**1**	1.006	1.004–1.010	1.007	1.003–1.012
**50**	1.372	1.201–1.609	1.421	1.167–1.804
**100**	1.881	1.442–2.589	2.019	1.362–3.255
**200**	3.539	2.079–6.705	4.078	1.854–10.593

Odds Ratios (OR) and 95% Confidence Intervals (CI) for creatinine adjusted urinary 8-oxodG levels were computed by the logistic regression procedure form the parsimonious model, which included only significant predictors (p < 0.05). APF refers to advanced periductal fibrosis, and CCA to cholangiocarcinoma.

*In the CCA risk model, age remained a significant predictor, therefore the OR and 95% CI that are reported are adjusted for age or aOR.

### Evaluating the performance of urinary 8-oxodG as a biomarker for advanced periductal fibrosis and cholangiocarcinoma

**Fig 3:**
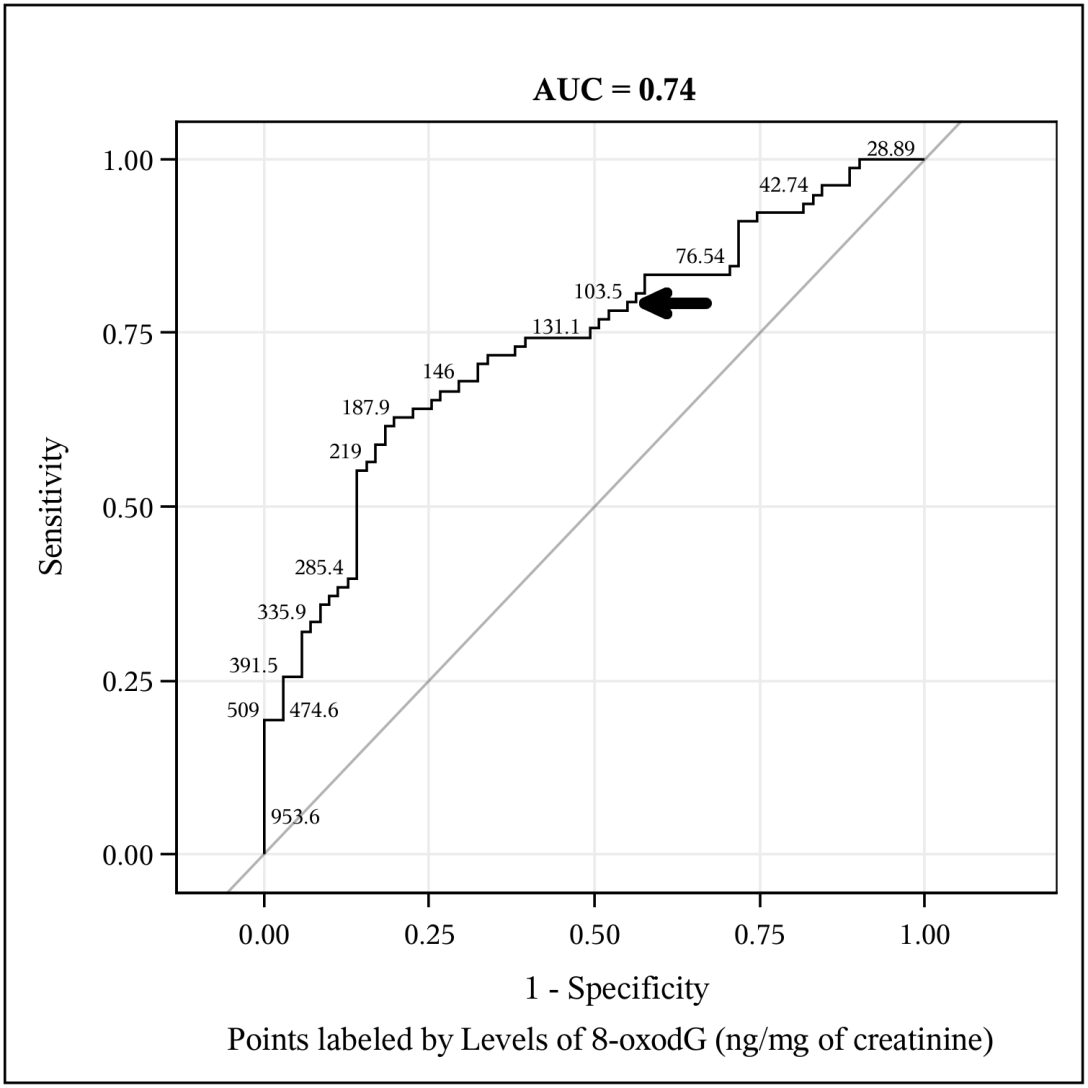
Levels of urine 8-oxodG detected by immunoassay can identify *O*.*viverrini* infected individuals with advanced periductal fibrosis (APF). The Receiver Operating Characteristic (ROC) curve illustrates the predictive power of urinary 8-oxodG in identifying APF+ individuals. The predictive power of the assay, measured by the area under curve (AUC) is 74%. The curve is labeled with selected actual amounts of 8-oxodG (ng/mg) detected in study participants; labeled points are presented, at random, in a way to maximize legibility. The model used to construct the ROC curve included negative controls (EN and APF- individuals, n = 71) and APF+ individuals (n = 78). The bold arrow points to the concentration of urine 8-oxodG identified as an optimal diagnostic threshold for corroborating APF status.

**Fig 4:**
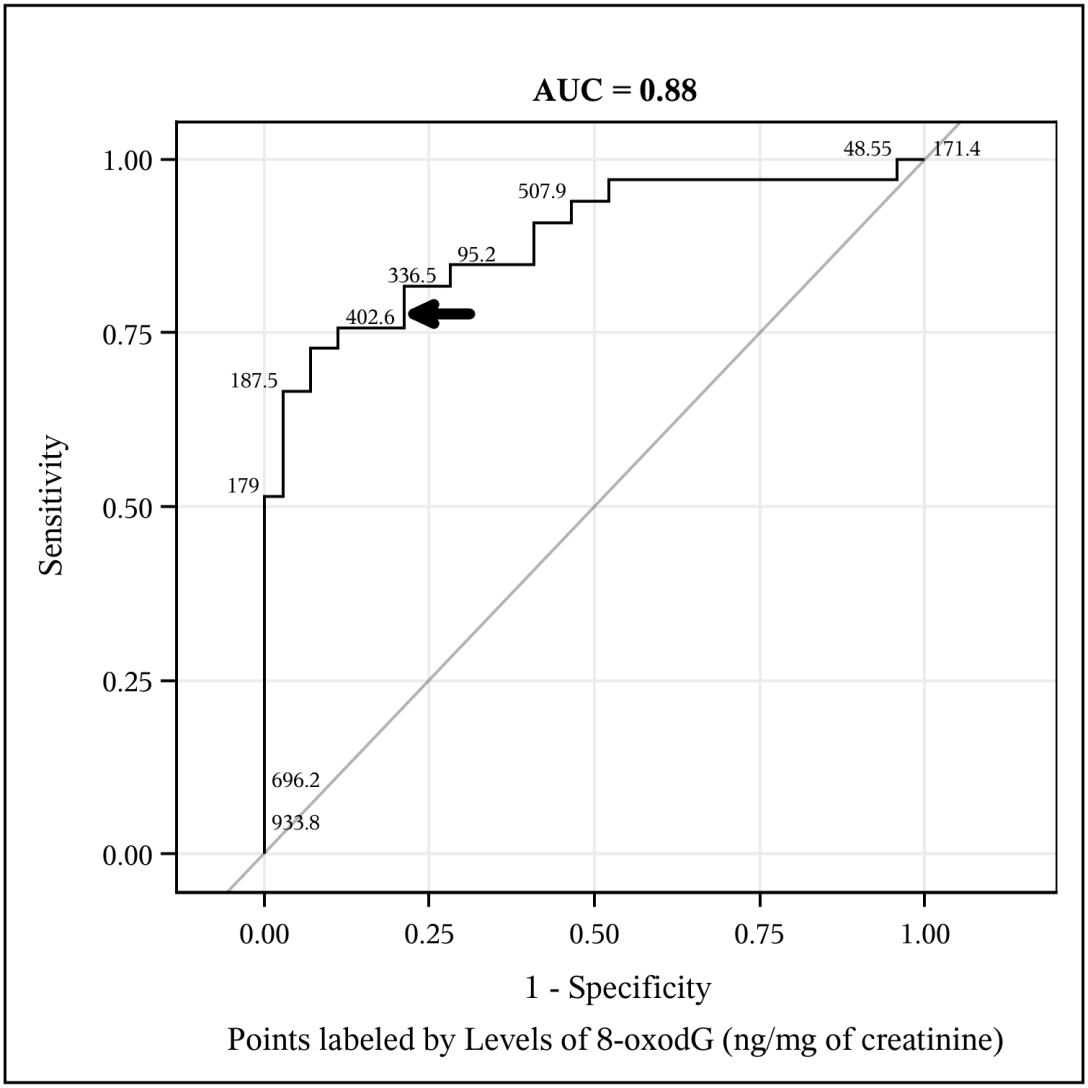
Levels of urine 8-oxodG detected by immunoassay can identify *O*.*viverrini* infected individuals with intrahepatic cholangiocarcinoma (CCA). The Receiver Operating Characteristic (ROC) curve illustrates the predictive power of urine 8-oxodG in identifying *O*. *viverrini*-induced CCA individuals. The predictive power of the assay measured by the area under curve (AUC) is 88%. The curve is labeled with selected actual amounts of 8-oxodG (ng/mg) detected in study participants; labeled points are presented, at random, in a way to maximize legibility. The model used to construct the ROC curve included negative controls (EN and APF- individuals, n = 71) and CCA individuals (n = 33). The bold arrow points to the concentration of urine 8 oxodG identified as an optimal diagnostic threshold for corroborating CCA status.

Receiver operating characteristic (ROC) curves constructed from levels of creatinine-adjusted urinary 8-oxodG measured in study participants are presented in Figs 3 and 4. The area under the ROC curve (AUC) describes the probability of correctly identifying a positive individual as a ‘case’ and a negative individual as a ‘non-case’. An AUC of 1 would describe a diagnostic test that would correctly identify all cases and all non-cases 100% of the time. The 45-degree line in the ROC plot marks the “chance diagonal”, which corresponds to an ROC curve with an AUC of 0.50 [50]. Figs 3 and 4 are labeled with the AUC values of each ROC curve; the ROC curve generated from a diagnostic model of APF+ individuals has an AUC of 0.74 and the AUC of the ROC generated from a diagnostic model of CCA individuals is 0.88. This means that levels of urinary 8-oxodG in urine correctly identifies individuals with *O*. *viverrini*-induced APF 74% of the time and correctly identifies individuals with *O*. *viverrini*-induced CCA 88% of the time. The diagnostic positivity thresholds were established as described in the methods section and are presented in Table 3 along with the relevant diagnostic validity parameters (sensitivity and specificity), the PPV, NPV, LR+, and LR- for the urinary 8-oxodG assay.

**Table 3:** Urinary 8-oxodG diagnostic positivity thresholds (DPT) and corresponding diagnostic validity parameters can be implemented in the clinical setting to detect advanced periductal fibrosis (APF) or cholangiocarcinoma (CCA) in individuals resident in *Opisthorchis viverinni* endemic areas.

	8-oxodG DPT (ng/mg creatinine)	Sensitivity	Specificity	PPV	NPV	LR+	LR-
**APF**	97	0.72	0.62	0.65	0.69	1.89	0.46
**CCA**	363	0.85	0.70	0.74	0.82	2.87	0.22

The assay validity parameters including diagnostic sensitivity, diagnostic specificity, PPV, NPV, LR+, and LR- were elucidated from the range of values modeled by the ROC curves in Figs 3 and 4. The process by which diagnostic positivity thresholds were identified is described in detail in the methods section of the manuscript. The values reported reflect the 'optimal' threshold levels identified from the logistic regression model and from the ROC curve analyses. The indicated diagnostic threshold concentrations of urinary 8-oxodG correctly identify APF+ individuals 72% of the time and CCA individuals 85% of the time.

## Discussion

*O*. *viverrini*-infected individuals with advanced periductal fibrosis (APF) as determined by abdominal ultrasound had markedly elevated levels of urinary 8-oxodG compared to *O*. *viverrini*-infected individuals without APF. Moreover, the concentrations of 8-oxodG in the urine of individuals with APF were comparable to the highly elevated levels of this oxidatively modified DNA lesion in individuals with *O*. *viverrini*-induced CCA [28, 31–33]. These results clearly suggest that elevated levels of this metabolite in urine are indicative of hepatobiliary fibrogenesis and tumorogenesis from chronic *O*. *viverrini* infection. Moreover, levels of 8-oxodG in the urine of *O*. *viverrini* infected individuals with APF or CCA individuals corroborated the ‘gold’ standard diagnostics used to detect both of these hepatobiliary pathologies in a dose-dependent manner: e.g., the highest 50 unit increment of 8-oxodG (200 units) indicated an increased risk of diagnosis of APF or CCA by 354% and 408%, respectively, compared to individuals with no detectable levels of urinary 8-oxodG. Furthermore, the risk models used to evaluate the utility of 8-oxodG as a biomarker were also used to identify the diagnostically relevant levels of 8-oxodG in the urine of study participants. The identification of these diagnostic threshold levels of urinary 8-oxodG, if corroborated in additional larger scale validation studies, would have important implications for urine 8-oxodG as diagnostic tool in field settings, with special significance in resource-limited settings, since they establish benchmarks that may be used to identify individuals at-risk of CCA and refer them for further testing (e.g., confirmatory abdominal ultrasound diagnosis) and preventive chemotherapy.

In order to assess the utility of 8-oxodG as a candidate biomarker for *O*. *viverrini*-induced APF and CCA, we also constructed a logistic regression risk model that initially included creatinine-adjusted urinary 8-oxodG levels along with significant demographic, behavioral, and immunological covariates associated with chronic *O*. *viverrini* infection. In this risk model, creatinine-adjusted urinary 8-oxodG emerged as the only significant predictor of APF, especially at higher concentrations of 8-oxodG. Creatinine adjusted 8-oxodG was also a significant predictor of CCA status when adjusted for age. Moreover, the risk model showed that 50 unit increases in creatinine-adjusted urinary 8-oxodG (in ng/mg) increased its ability to corroborate APF status by 137% and CCA status by 142% (after adjusting for age) and also helped to establish diagnostically relevant threshold levels of this biomarker. Moreover, elevated levels of urine 8-oxodG accurately identified progression to advanced stages of the disease (Figs 3 and 4), with high odd ratios (ORs) of APF and CCA associated with higher levels of this candidate biomarker (Table 2). As APF is precursor stage to CCA [36], a simple, non-invasive assay for 8-oxodG in urine as a biomarker for this *O*. *viverrini*-induced pathology would be of profound benefit in Southeast Asia, especially among populations residing in the resource-limited settings of the Mekong Basin region, where the incidence of intrahepatic CCA is the highest in the world [51].

The methods and standards for measuring urinary 8-oxodG have received considerable attention[24, 40], with a consensus on using urine 8-oxodG as a biomarker established by the European Standards Committee on Urinary (DNA) Lesion Analysis (ESCULA) [24]. When properly detected, measured, and analyzed, ESCULA determined urine 8-oxodG to be a robust, accurate, reproducible, and (“remarkably”) stable urine biomarker for OS [24]. The production of 8-hyrdroyguanine is almost exclusively elicited by OS, with the main attack site by oxidative radicals at the N7-C8 bond [26, 52, 53]. While there are findings that suggest that diet contributes to urinary levels of thymine glycol and 8-oxo-7,8-dihydro-guanine (8-oxoGua) [40], little or no information has been reported that this applies to urinary levels of oxidatively modified 2′-deoxyribonucleosides (8-oxodG), hence obviating diet as a potential confounder. As with other studies, the present findings showed a relatively strong association between levels of urinary creatinine and levels of urinary 8-oxodG, which (as explained by Barregard et al. [40]) are likely due to differences in body mass index (BMI), since metabolic rate is associated with lean body mass and a higher metabolic rate generates a larger amount of modified 2-deoxyribonucleoside products in urine [40]: e.g., in general, males excrete more 8-oxodG than females per kilogram body weight [40]. Hence, following the recommendations of Barregard et al. [40] for using 8-oxodG in cross-sectional studies, we normalized urinary 8-oxodG concentrations by individual creatinine concentrations as determined by the Jaffe method [38].

Oxidative DNA damage as a key event in *O*. *viverrini*-induced CCA has been studied extensively in an animal model (see [23] for review), where oxidatively damaged DNA bases formed along the inflamed intrahepatic biliary ducts, at sites adjacent to the parasite, perhaps from persistent wound repair [31, 32]. Immunohistochemical studies in a hamster model of *O*. *viverrini*-induced CCA showed that inflammatory cells surrounding the parasite in the bile duct, such as mononuclear cells and eosinophils, generate reactive oxygen species (ROS), which induce oxidative stress (OS) and increased cleavage of 8-oxodG [31, 32]. Additionally, immunohistochemical analyses on livers resected from humans with *O*. *viverrini*-induced CCA showed 8-oxodG in tumor tissue from the bile duct [20, 33]. Moreover, Thana et al. [33] observed elevated levels of 8-oxdG in the urine of individuals infected with *O*. *viverrini* compared to healthy controls as also observed in the current study (Fig 2 Panel A). However, the current study adds to the literature the observation that urine 8-oxodG can be detect in markedly higher concentration when *O*. *viverrini* infected individuals who have progressed to APF or CCA (Fig 2).

The observation that urinary 8-oxodG concentrations were similar in APF and CCA individuals support our hypothesis that common mechanisms drive bile duct fibrosis and bile duct tumorogenesis from chronic *O*. *viverrini* infection [8–10, 12, 13]. It is also in keeping with findings from other groups that chronic bile duct inflammation leads to a desmoplastic stroma (i.e., fibrosis) in the bile duct that precedes CCA (reviewed by Sirica and Gores [36]. As depicted in S1 Fig, elevated levels of 8-oxodG in the urine of APF individuals reflects ongoing tissue repair and the smoldering inflammatory milieu” [14] in the hepatobiliary epithelia from persistent injury from the parasite [15, 30, 54, 55]. This “desmoplastic reaction” to chronic *O*. *viverrini* infection provides a rich niche for cancer cells to develop and progress [1, 36, 56, 57]. Currently, therapeutic targeting to reduce desmoplastic stroma (periductal fibrosis) to prevent CCA is being investigated [36, 56, 57].

Taken together, these data show that urine 8-oxodG may be an excellent biomarker for the advanced hepatobiliary pathology that occurs prior to *O*. *viverrini*–induced CCA. In keeping with a recent position statement from the European Group on Tumor Markers [58], the current manuscript places urine 8-oxodG as a “candidate biomarker” for APF and CCA in the “discovery phase”, i.e., “when differential expression of a specific marker is shown to associate with a ‘gold’ standard clinical outcome”. The next phase of biomarker development for urine 8-oxodG is the “verification stage”, when our analyses would be extended to a much larger sample (i.e., hundreds) of individuals infected with *O*. *viverrini* and at risk of CCA. The objective of the verification stage would be to incorporate the broadest range of cases and controls in order to capture the environmental, genetic, biological, and stochastic variation in the population in the Mekong Basin Subregion. As the diagnostic sensitivity of the candidate biomarker has been established herein, the verification stage would focus on the specificity of the candidate biomarker and the utility of the diagnostic threshold levels set forth in this manuscript [58]. If validated, levels of urinary 8-oxodG may be an inexpensive way to identify at-risk individuals, who may be referred for subsequent, more demanding testing. This process would streamline diagnostics for APF and CCA and perhaps improve the utilization of the limited resources allocated to cancer screening in this region.

## Conclusion

The findings herein confirm previous observations that severe hepatobiliary disease occurs early and asymptomatically among residents in *O*. *viverrini* endemic areas. A simple, non-invasive assay targeting 8-oxodG in urine would be of profound benefit to populations in Southeast Asia, especially in the resource-limited settings of the Mekong Basin region countries of Thailand, Laos and Cambodia, where the incidence of *O*. *viverrini*-induced CCA is the highest in the world [7]. The future plan for the candidate biomarker includes moving to a verification step to test its accuracy in a larger sample size [58].

## Supporting Information

S1 Fig 8-oxodG is a biomarker of disease progression in individuals chronically infected with *Opisthorchis viverrini*. An illustration of the effect of chronic inflammatory challenge experienced by OV-infected individuals and its relationship to oxidative stress as determined by levels of 8-oxodG in urine. Increases in the levels of creatinine-adjusted 8-oxodG in urine are biomarkers for the risk of advanced periductal fibrosis (APF) and cholangiocarcinoma (CCA).
(TIF)

S1 Text Supplementary equations and definitions. This file includes equations describing the logistic regression models used in this manuscript, equations for computing assessment measures of diagnostic accuracy of the urine 8-oxodG diagnostic assay, as well as definitions of relevant epidemiologic terminology used in evaluating assay performance.
(DOCX)
