# Supplementary figures and images for: Levels of 8-OxodG Predict Hepatobiliary Pathology in Opisthorchis viverrini Endemic Settings in Thailand
Source: PLoS Negl Trop Dis. 2015 Jul 31;9(7):e0003949. doi: 10.1371/journal.pntd.0003949 (PMC4521778; doi:10.1371/journal.pntd.0003949)

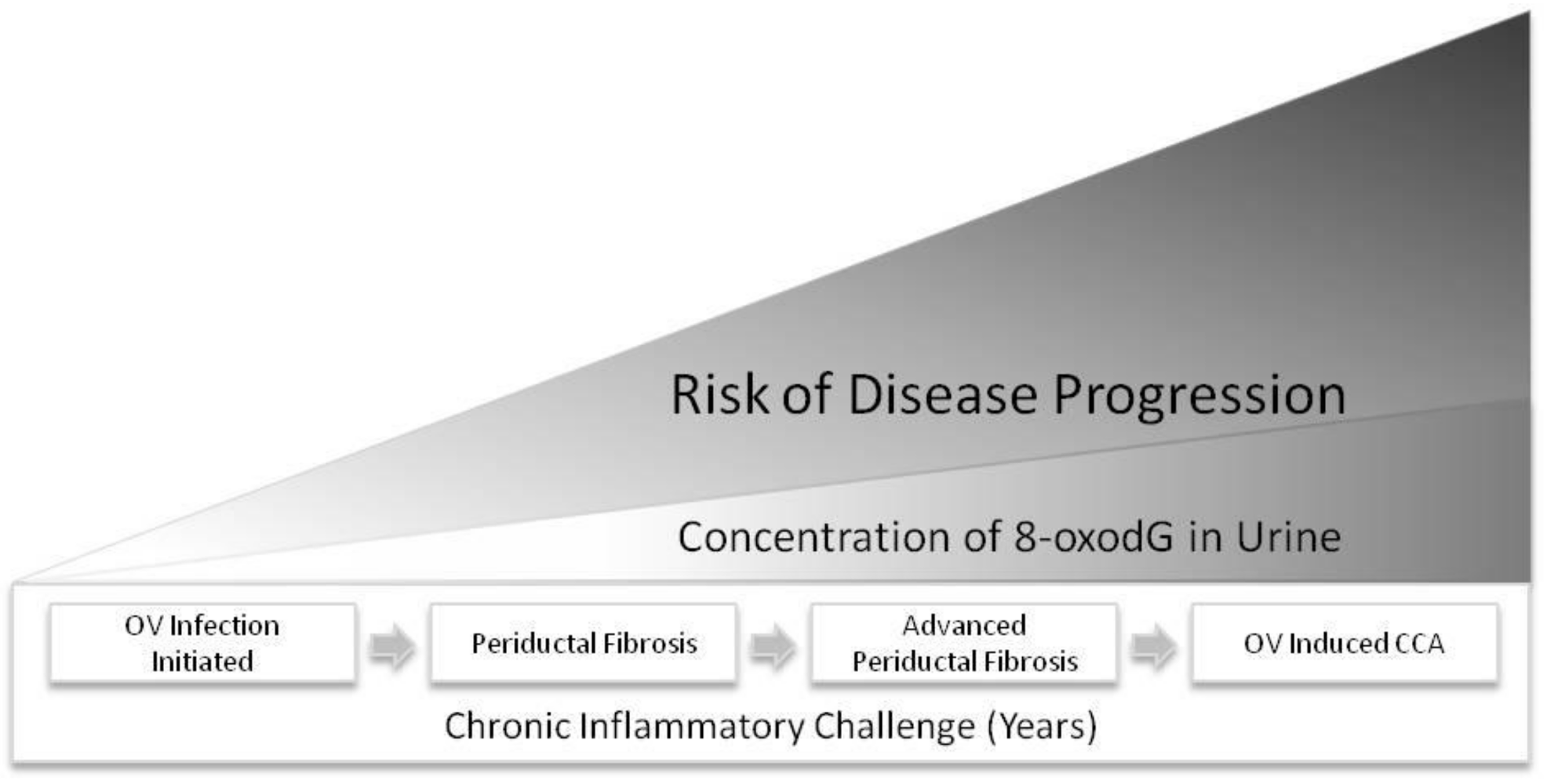

Supplement: S1 Fig — An illustration of the effect of chronic inflammatory challenge experienced by OV-infected individuals and its relationship to oxidative stress as determined by levels of 8-oxodG in urine. Increases in the levels of creatinine-adjusted 8-oxodG in urine are biomarkers for the risk of advanced periductal fibrosis (APF) and cholangiocarcinoma (CCA). (TIF) [file pntd.0003949.s001.tif]
