# Supplementary material for: Levels of 8-OxodG Predict Hepatobiliary Pathology in Opisthorchis viverrini Endemic Settings in Thailand
Source: PLoS Negl Trop Dis. 2015 Jul 31;9(7):e0003949. doi: 10.1371/journal.pntd.0003949 (PMC4521778; doi:10.1371/journal.pntd.0003949)
Supplement: S1 Text — This file includes equations describing the logistic regression models used in this manuscript, equations for computing assessment measures of diagnostic accuracy of the urine 8-oxodG diagnostic assay, as well as definitions of relevant epidemiologic terminology used in evaluating assay performance. (DOCX) [file pntd.0003949.s002.docx]

**S1 Text - Supplementary Equations and Definitions**

**Logistic regression models**

**Possible predictor variables considered in the initial logistic regression model.** The initial stages of model development included all potentially relevant variables. Smoking was not considered in the model of CCA individuals, because this information was not available for this group of participants.

 (1)

**The final parsimonious models comparing control individuals to either APF+ or CCA individuals.** During this second stage of model development, the only significantly relevant biological predictor retained was urinary 8-oxodG. Age remained a significant predictor in the model of CCA individuals, but was not retained in the model of APF+ individuals as it was no longer significant.

$\text{predicted logit}\left(\text{status}=1\right)=\alpha+\beta_{1}*\left(\text{levels of 8-oxodG in urine}\right)+\beta_{2}*\left(\text{age}\right)$ (2)

**Measures used to evaluate the performance of the diagnostic assay**

**Assessing accuracy of the urinary 8-oxodG diagnostic assay.**

$\text{Positive Predictive Value (PPV) = }\frac{\text{sensitivity}*\text{prevalence}}{\text{sensitivity}*\text{prevalence}+\left(1-\text{specificity}\right)*\left(1-\text{prevalence}\right)}$ (3)

$\text{Negative Predictive Value (NPV) = }\frac{\text{specificity}*\left(1-\text{prevalence}\right)}{\left(1-\text{sensitivity}\right)*\text{prevalence}+\text{specificity}*\left(1-\text{prevalence}\right)}$ (4)

**Definitions**

Sensitivity is defined as “the proportion of observations correctly classified" as having advanced hepatobiliary disease in the form of either APF or CCA or true positive probability [[50](#_ENREF_50)].

Specificity is defined as “the proportion of observations correctly classified as 'normal' or individuals who are either endemic normal (negative for *O. viverrini* infection) or *O. viverrini* infected but APF negative” [[50](#_ENREF_50)] or true negative probability. The term 1-specificity describes the proportion of observations incorrectly classified as 'events' or a false positive.

The LR+ is an index of diagnostic accuracy and describes the increased likelihood of observing a positive test result in patients with the disease.

LR- describes the likelihood, after subtracting from 1, of observing a negative test result in individuals without the disease.
